# Editorial: Advanced healthcare biomaterials for surface functionalization and controlled drug delivery

**DOI:** 10.3389/fbioe.2023.1309003

**Published:** 2023-10-19

**Authors:** Gianina Popescu-Pelin, Anita Ioana Visan, Muhammad Arif Mahmood, Marwan Khraisheh

**Affiliations:** ^1^ National Institute for Laser, Plasma and Radiation Physics, Magurele, Romania; ^2^ Missouri University of Science and Technology, Rolla, MO, United States; ^3^ Texas A&M University at Qatar, Doha, Qatar

**Keywords:** controlled drug delivery, surface functionalization, healthcare biomaterials, hydrogel systems, drug-loaded materials

The field of healthcare has witnessed remarkable progress in recent years, particularly in the area of advanced biomaterials for surface functionalization and controlled drug delivery. The editorial aims to highlight the key contributions to this Research Topic, shedding light on the design and synthesis of biomaterials, their impact on drug delivery, and their potential clinical applications.

Conventional drug delivery methods often suffer from fluctuating drug concentrations, leading to potential side effects and reduced therapeutic efficacy. To address these challenges, researchers have developed approaches for controlled drug release, such as sustained-release technologies. These technologies involve the use of biomaterials that can release drugs over an extended period, reducing dosing frequency and improving patient outcomes.

One of the articles focused on the use of hydrogel systems for targeted cancer therapy Li et al. It was demonstrated that the high hydrogels’ drug-carrying capacity and controlled drug release capabilities are ideal for delivering various substances such as chemotherapeutic drugs, radionuclides, immunosuppressants, hyperthermia agents, and phototherapeutic agents. They can trigger cascade reactions and respond intelligently to environmental changes, allowing for targeted drug delivery to specific locations. Some challenges such as inefficient and expensive synthesis hinder the adaptability; however, further research may revolutionize cancer treatment and improve outcomes for patients. The hydrogels can also be applied to repair and regenerate various defected tissues including bone, skin, muscle, and nerve tissues Li et al. For instance, this technique can be applied to repair cartilage defects. It introduces the principles of developing hydrogels for cartilage regeneration and the criteria for optimized outcomes, such as biocompatibility, cell affinity, and tissue integration properties. In order to produce hydrogels for cartilage repair, various materials, including synthetic polymers, polysaccharides, proteins, and peptides can be utilized Li et al. Hydrogels are also important for providing the biophysical and biochemical factors in regulating the fate of stem cells, thus enhancing tendon regeneration. To excel in this field, there are various challenges that need to be addressed with a careful consideration of various factors, including biocompatibility, cell affinity, tissue integration properties, feasibility, and the choice of materials.

Another promising approach for cancer treatment is presented by Zhang et al. It was identified that the photodynamic therapy (PDT) for oral squamous cell carcinoma (OSCC) can be attained using manganese-doped carbon dots (Mn-CDs). Mn-CDs characterizations confirmed the successful synthesis of Mn-CDs and provided insights into their morphology, size, surface charge, chemical bonds, crystal structure, and functional groups. These Mn-CDs demonstrated excellent photodynamic efficiency and satisfactory biosafety, both *in vitro* and *in vivo*, indicating their potential as a novel therapeutic agent for cancer treatment.

The study of Zhang et al. also demonstrated that vancomycin-loaded silk fibroin microspheres in the injectable hydrogel system effectively deliver vancomycin, leading to improved antibacterial activity and enhanced bone regeneration compared to conventional treatment approaches Zhang et al. Silk fibroin microspheres were utilized as a carrier for vancomycin, a potent antibiotic commonly used to treat osteomyelitis. The microspheres were loaded with vancomycin and then incorporated into an injectable hydrogel system. The combination of the microspheres and hydrogel provided sustained release of vancomycin, allowing for prolonged and localized drug delivery at the site of infection. *In vitro* studies involved evaluating the release kinetics of vancomycin from the microspheres within the hydrogel, assessing antibacterial activity against relevant pathogens, and evaluating the biocompatibility of the system. *In vivo* studies comprised the implantation of the vancomycin-loaded silk fibroin microspheres in the injectable hydrogel system into an osteomyelitis model. The researchers assessed the therapeutic efficacy of the treatment by monitoring bacterial clearance, bone regeneration, and inflammation reduction.

The article titled “*Zwitterionic coating assisted by dopamine with metal-phenolic networks loaded on titanium with improved biocompatibility and antibacterial property for artificial heart*” focused on the development of a surface coating for titanium-based artificial heart implants that enhances biocompatibility and antibacterial properties Meng et al. The study employed a combination of polydopamine and poly-(sulfobetaine methacrylate) (SBMA) to form a coating on the surface of titanium substrate. The coating process was initiated by copper ions (Cu^2+^). The research presented a zwitterionic coating assisted by dopamine with metal-phenolic networks loaded on titanium, improving the biocompatibility and antibacterial properties of artificial heart implants. The coating demonstrated effective antibacterial activity, antiplatelet adhesion properties, and stability in different environments. These findings have implications for the development of optimized surfaces for titanium-based substrates in artificial heart implants, with the potential to reduce the need for long-term prophylactic antibiotics and anti-thrombotic drugs.

The study of Escobar et al. focused on the functionalization of breast implants using cyclodextrin *in-situ* polymerization as a local drug delivery system for augmentation mammaplasty. The article emphasized the need for surface modification techniques to overcome limitations and complications associated with silicone implants, such as the capsular contracture formation. Various drugs and polymers were explored to improve the performance of silicone implants and reduce complications. The polymerization process, optimization parameters, and the potential of cyclodextrin-based coatings for controlled drug release were also discussed. The research also highlighted the broader applications of cyclodextrin-based coatings in drug delivery systems and the need for further research to overcome complications associated with silicone implants.

In conclusion, the Research Topic on advanced healthcare biomaterials for surface functionalization and controlled drug delivery encompasses various scientific and technological advancements. From the design and synthesis of biomaterials to their clinical and commercial impact, researchers have made significant progress in improving drug delivery strategies and therapeutic outcomes. By harnessing the potential of designing and engineering biomaterials through advanced manufacturing and characterization techniques, healthcare professionals can revolutionize the field of medicine and improve the lives of countless individuals.

